# The Role of 11C-Methionine PET Imaging for the Evaluation of Lymphomas: A Systematic Review

**DOI:** 10.3390/hematolrep16040072

**Published:** 2024-11-27

**Authors:** Francesco Dondi, Maria Gazzilli, Gian Luca Viganò, Antonio Rosario Pisani, Cristina Ferrari, Giuseppe Rubini, Francesco Bertagna

**Affiliations:** 1 Nuclear Medicine, Università Degli Studi di Brescia and ASST Spedali Civili di Brescia, 25123 Brescia, Italy; francesco.bertagna@unibs.it; 2 Nuclear Medicine, ASL Bari—P.O. Di Venere, 70012 Bari, Italy; marinagazzilli@msn.com; 3 Clinical Engineering, ASST Spedali Civili di Brescia, 25123 Brescia, Italy; gianluca.vigano@asst-spedalicivili.it; 4 Section of Nuclear Medicine, Interdisciplinary Department of Medicine, University of Bari “Aldo Moro”, Piazza Giulio Cesare 11, 70124 Bari, Italy; antoniorosario.pisani@uniba.it (A.R.P.); cristina.ferrari@uniba.it (C.F.); giuseppe.rubini@uniba.it (G.R.)

**Keywords:** PET, PET/CT, positron emission tomography, DLBCL, lymphoma, 11C-methionine, central nervous system

## Abstract

**Background**: In the last years, different evidence has underlined a possible role for [11C]-methionine ([11C]MET) positron emission tomography (PET) imaging for the evaluation of lymphomas. The aim of this paper was, therefore, to review the available scientific literature focusing on this topic. **Methods**: A wide literature search of the PubMed/MEDLINE, Scopus and Cochrane Library databases was conducted in order to find relevant published articles investigating the role of [11C]MET in the assessment of lymphomas. **Results**: Eighteen studies were included in the systematic review and the main fields of application of this imaging modality were the evaluation of disease, therapy response assessment, prognostic evaluation and differential diagnosis with other pathological conditions. **Conclusion**: Even with heterogeneous evidence, a possible role for [11C]MET PET imaging in the assessment of lymphomas affecting both the whole body and the central nervous system was underlined. When compared to [18F]fluorodesoxyglucose ([18F]FDG) imaging, in general, similar results have been reported between the two modalities in these settings.

## 1. Introduction

Lymphomas and hematological malignancies are a heterogeneous group of diseases that affect a significant number of patients worldwide. For these subjects, diagnosis and treatment are crucial to establish a clear and specific therapeutic regimen that could, therefore, impact their prognosis [1,2,3,4].

Positron emission tomography (PET) is an imaging modality that, in recent years, has demonstrated its added value in the assessment of a wide variety of different conditions, both neoplastic or benign [5,6,7,8,9]. In this setting, since 18F-fluorodeoxyglucose ([18F]FDG) has the ability to reflect the glycolytic activity of tissues, it is the tracer mainly used for positron imaging in order to assess the presence of and characterize different pathological conditions. In particular, [18F]FDG is able to enter the cells using glucose transporter 1 (GLUT1) and being phosphorylated by hexokinase to [18F]FDG-6 phospate ([18F]FDG-6P). [18F]FDG-6P is, therefore, trapped in the cell and, owing to a very low concentration of glucose-6-phosphate, the reverse reaction does not take place. As a consequence, [18F]FDG is used to assess the glucose uptake of cells [10]. In this setting, the role of [18F]FDG PET imaging for the initial assessment, the post-therapeutic evaluation and its prognostic value have been clearly demonstrated for both Hodgkin’s lymphoma (HL) and non-HL (NHL), such as diffuse large B-cell lymphoma (DLBCL) or follicular lymphoma, but also for other hematological conditions such as leukemias [11,12,13,14,15,16,17,18,19,20,21].

In recent years, however, different PET tracers, able to image different metabolic pathways of different tissues, have emerged and are proving their value in a wide range of pathological conditions [8,22,23,24,25,26,27]. As a consequence, hematological malignancies have also been studied with different tracers [20,28,29,30]. [11C]-methionine ([11C]MET) is a radiolabeled amino acid essential for different biological pathways that take place in cells, such as the synthesis of protein, the conversion to the predominant biologic methyl group donor S-adenosylmethionine, polyamine synthesis and the transsulfuration pathway. In this setting, [11C]MET uptake reflects increased amino acid intake and protein synthesis by the cells; it is positively related to cellular proliferation activity and, therefore, avid uptake of this precursor is a normal feature of rapidly proliferating tissues [31]. Interestingly, neoplastic cells are dependent on the external supply of methionine and the transmethylation rate is higher in tumor cells, with a difference in the rates of uptake of amino acids between neoplastic and non-neoplastic cells that may be 2.5–3.5 fold higher [32,33,34,35,36]. Furthermore, the uptake of methionine primarily reflects transmembrane transport by the sodium-independent L-transporter into cells. This transport is driven by the concentration gradient and is, thus, influenced by the intracellular metabolism of the amino acid, which, in turn, reflects proliferation activity [31]. In this setting, there is, therefore, a clear difference in the mechanisms of action of [18F]FDG and [11C]MET: As mentioned, the first radiotracer is able to reflect the glycolytic activity of the cells, while the second radiopharmaceuticals can give information on the protein synthesis of the tissues.

In addition to its ability to image the ratio of protein synthesis of tissues, [11C]MET offers an interesting advantage if compared to [18F]FDG, that is, its ability to visualize neoplastic lesions in anatomic territories not clearly evaluable by assessing their glycolytic activity because of the presence of physiological high uptake in normal tissues, such as in the brain [31,37]. In this setting, different evidence in the literature has demonstrated that [11C]MET PET can be useful for the assessment of different types of tumor and this tracer has been particularly studied for neoplasms of the brain [38]. Interestingly, inflammatory processes and benign diseases have also shown the possibility to be positive when imaged with this radiopharmaceutical [31,39]. In addition, evidence in the literature has demonstrated that [11C]MET can accumulate in most lymphomas and other hematological conditions [40,41].

The aim of this review is, therefore, to provide an overview of the existing literature on the value of [11C]MET PET imaging in the assessment of lymphomas.

## 2. Materials and Methods

This systematic review was performed according to the “Preferred Reporting Items for a Systematic Review and Meta-Analysis” (PRISMA 2020 statement), employed as a guide in its development. Pre-registering was not carried out while the review was registered in the Open Science Framework database (https://osf.io/tke8q, accessed on 6 November 2024).

### 2.1. Search Strategy

A wide literature search of the PubMed/MEDLINE, Scopus and Cochrane Library databases was performed to identify published articles addressing the role of [11C]MET PET imaging in the assessment of lymphomas. The algorithm used for the research was: “11C-methionine AND (‘pet’/exp OR pet) AND lymphoma”.

The search had no beginning date limit and it was updated until 15 July 2024. Only articles in the English language were considered. Preclinical studies, conference proceedings, reviews, editorials or original papers with only a patient affected by lymphoma and imaged with [11C]MET were not included in the review. The references of the retrieved articles were also screened for additional papers to expand our search (Table 1).

### 2.2. Study Selection

Two researchers (F.D. and F.B.) independently reviewed the titles and abstracts of the retrieved articles. The same two researchers then independently reviewed the full-text version of the identified articles to determine their eligibility for the inclusion.

### 2.3. Quality Assessment

The quality assessment of these studies, including the risk of bias and applicability concerns, was carried out using Quality Assessment of Diagnostic Accuracy Studies version 2 (QUADAS-2) evaluation [42]. Quality assessment was performed independently by two reviewers.

### 2.4. Data Extraction

Two reviewers independently evaluated the retrieved studies to collect relevant information. For each study included in the review, data concerning the basic information of the study such as first author name, year of publication, country of origin, design of the study, radiopharmaceutical used, number of patients and type of lymphoma were collected. Furthermore, information about the type of PET tomograph used, the activity of the injected tracer, the type of imaging analysis used, the setting of the study and the main results were also collected. The main findings of the articles included in this review are reported in the Results section.

## 3. Results

### 3.1. Literature Search

**Figure 1:**
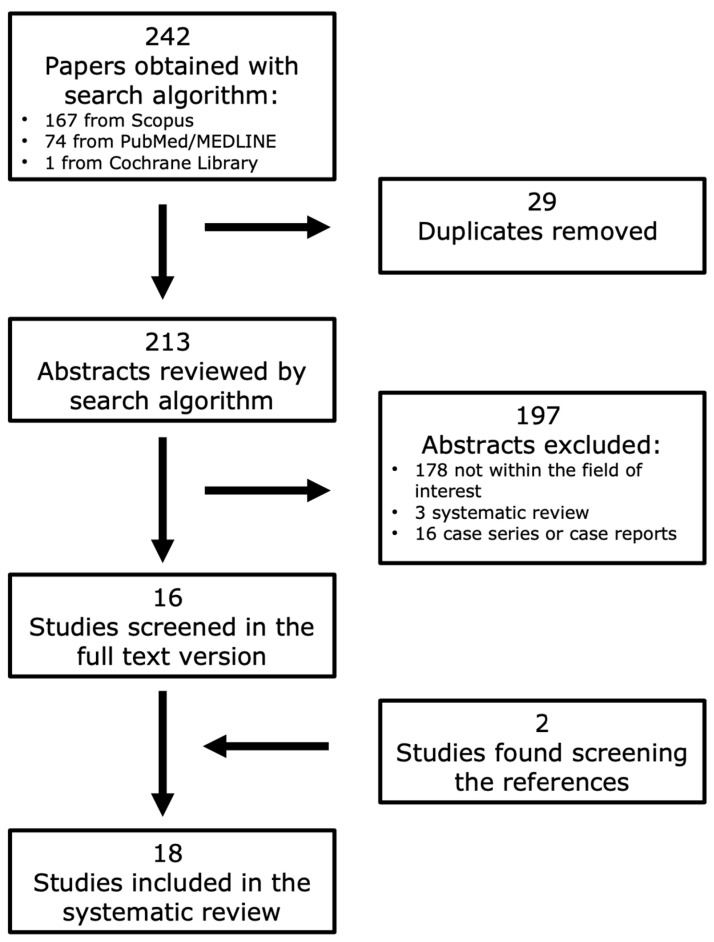
Flowchart of the research of eligible studies evaluating the role of [11C]MET PET imaging in the assessment of lymphomas.

The literature search retrieved a total of 242 papers: 167 from Scopus, 74 from PubMed/MEDLINE database and 1 from the Cochrane Library. After removing the duplicates, a total number of 213 articles were obtained. After reviewing the titles and abstracts, 197 of them were excluded for different reasons: 178 because the reported data were not within the field of interest of this review, 3 were systematic reviews and 16 were care series or case reports. As a consequence, 16 studies addressing the role of [11C]MET PET imaging for the assessment of lymphoma were selected and retrieved in the full-text version [43,44,45,46,47,48,49,50,51,52,53,54,55,56,57,58]. Two additional studies were found after analyzing the references lists of these articles [59,60]. The total number of studies included in the review was, therefore, 18 (Figure 1).

**Figure 2:**
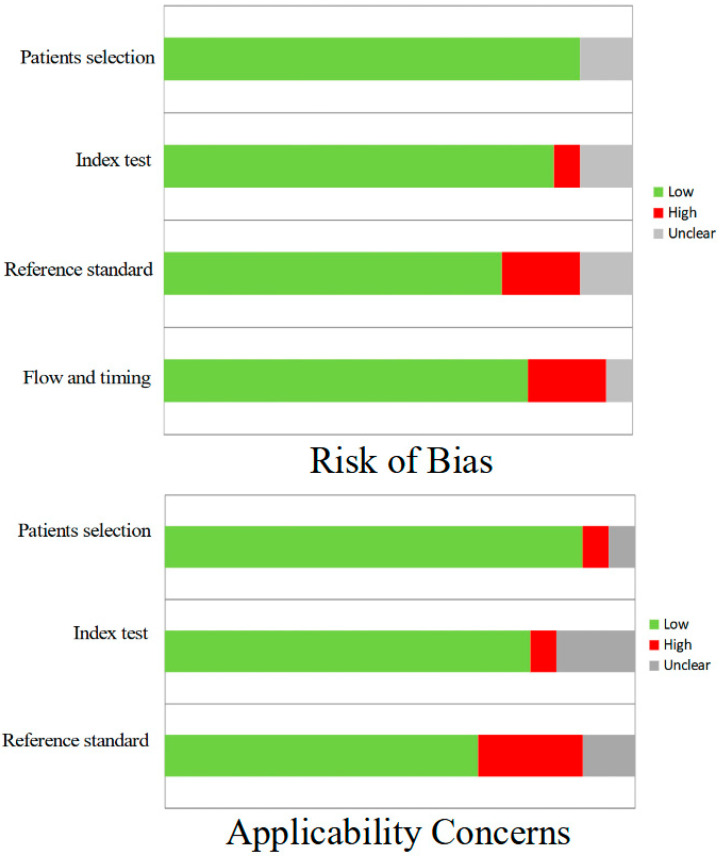
QUADAS-2 quality assessment for risk of bias and applicability concerns for the studies considered in the review.

In general, the quality assessment using QUADAS-2 evaluation underlined the presence of low risk of bias in most of the domains for all the studies included in the review (Figure 2).

Among the total number of studies included in the systematic review, 10 were retrospective [47,49,50,51,52,56,57,58,59,60]; seven had a prospective design [43,44,46,48,53,54,55], whereas in one case, it was not specified the nature of the research [45]. In terms of radiopharmaceuticals used, eight studies were performed using only [11C]MET [44,45,47,51,54,57,59,60], nine studies used both [11C]MET and [18F]FDG [43,46,48,49,50,52,53,56,58], while in one study, [11C]MET and [18F]fludarabine ([18F]FLUDA) were used [55]. Moreover, six studies were performed using a PET/computed tomography (CT) tomograph [53,54,55,57,58,60], 11 studies were performed with PET tomographs [43,44,45,46,47,48,49,50,51,52,59] and in a single case, the type of the tomograph was not specified [56]. The main characteristics of the studies and their results are briefly presented in Table 2 and Table 3.

### 3.2. Role of [11C]MET PET Imaging for the Evaluation of Lymphoma

The role of [11C]MET PET imaging for the assessment of patients affected by lymphomas has been investigated in different papers. First, Leskinen-Kallio et al. [43] evaluated the uptake of [11C]MET by NHL before therapy, revealing that the tracer accumulated in 13/14 subjects, with the exception of a patients that had an intermediate grade disease in the eyelid with the physiological accumulation of [11C]MET in the lacrimal gland that may have impaired its detection. Compared to [18F]FDG, the uptake rate of [11C]MET was higher (*p* < 0.01); however, the pattern of the two tracers was generally homogeneous, with the exception of two high-grade lymphomas that had heterogeneous uptake. Additionally, the tumor-to-plasma ratios increased faster for high- and intermediate-grade lymphomas for both tracers, even though considerable overlap was present for low-grade diseases. The uptake rate of [11C]MET tended to be higher in lymphomas with a large fraction of cells in the S-phase (r 0.62), while [18F]FDG uptake correlated poorly with this parameter (r 0.29). Interestingly, it was reported that, in the cluster analysis, [18F]FDG seemed to be better in distinguishing between high and other grades of lymphoma.

Similarly, Rodriguez et al. [46] evaluated the role of both [18F]FDG and [11C]MET PET imaging in predicting the malignancy grade of NHL. All tumors were visible with both tracers but no significant differences in terms of standardized uptake value (SUV), transport rate and mass influx were demonstrated between high- and low-grade lymphomas for [11C]MET (*p* value 0.9, 0.2 and 0.1, respectively). In contrast, for [18F]FDG, all the parameters were significantly different.

Nuutinen et al. [47] revealed that 31/32 lymphomas had increased [11C]MET uptake, reporting therefore a sensitivity of PET of 97%, without false positive findings. Median SUV was 6.6 (1.9–12.4) while the median fractional rate of tracer transport and methylation per unit time (K_i_) was 0.116 min−1 (0.025–0.201); no quantitative differences for primary and recurrent lymphomas could be established. In addition, [11C]MET K_i_ demonstrated the ability to distinguish between high and other grade NHL (*p* < 0.001), with a median SUV of 7.0 (5.4–12.4) in high-, 6.2 (1.9–10.4) in intermediate- and 5.7 (3.8–8.3) in low-grade disease, respectively. Additionally, the median K_i_ value was 0.162 min−1 (0.147–0.197) in high-, 0.099 (0.025–0.152) in intermediate- and 0.078 (0.056–0.152) in low-grade lymphomas, respectively. Focusing on HL, an SUV similar to that of high-grade NHL was reported and, additionally, lymphomas with a high K_i_ value tended to have a higher amount of cells in the S-phase (r2 0.46, *p* value 0.043).

Sutinen et al. [48] investigated the role of [11C]MET PET imaging in the staging of lymphomas, reporting that 55/178 lymph node regions were classified as diseased by both [18F]FDG and CT, and that 54/178 were classified as diseased by both [11C]MET and CT. Additionally, 11 nodal regions that CT showed to be normal were positive at both [18F]FDG and [11C]MET. PET would have upstaged the disease in three patients with both tracers, while [11C]MET would have downstaged the disease in one subject. In general, both radiopharmaceuticals seemed to accumulate avidly in both low- and high-grade disease and no clear and distinct differences in terms of lesion visualization could be shown. The authors concluded that the two imaging modalities seemed to be comparable in the detection of lymphomas; however, the physiological accumulation of [11C]MET hampered the interpretation of the images more, while it could be preferable in the staging setting in hyperglycemic patients.

An interesting paper by Kaste et al. [53] compared [18F]FDG and [11C]MET PET/CT in the assessment of pediatric lymphomas. At staging, 14/17 regions revealed concordant uptake of both tracers, while three nodal groups (Waldeyer’s ring, paraaortic region and liver) showed discordant metabolic activity. Additionally, intense [11C]MET uptake was demonstrated in the pancreas and in the liver, interfering, therefore, with disease detection in these regions. The number of abnormal sites of metabolic activity was nearly identical between the two imaging modalities, with the exception of nearly one third more foci being identified with [18F]FDG.

Focusing on the diagnostic ability of [11C]MET PET in primary central nervous system lymphoma (PCNSL) with atypical magnetic resonance imaging (MRI) appearance, Kawai et al. [49] revealed that all five patients with typical PCNSL showed a strong tracer uptake, while 2/4 subjects with atypical forms showed a weak and irregular pattern, and the other two only had faint uptake. Similarly to [18F]FDG, [11C]MET SUVmax was significantly higher in typical forms compared to atypical forms (SUVmax 5.9 ± 1.8 and 2.3 ± 0.3, respectively, *p* < 0.01). In addition, some kinetics parameters at [18F]FDG imaging were significantly different between typical and atypical forms.

Lastly, a comparison of the diagnostic ability of [18F]FDG and [11C]MET PET imaging in PCNSL was also performed by Kawase et al. [50]. At visual analysis, all PCNSL had [11C]MET uptake with a 100% sensitivity, overlapping the one of [18F]FDG. At semiquantitative analysis, normal brain tissue had lower uptake at [11C]MET compared to [18F]FDG (*p* < 0.002) and mean SUV of PCNSL at [11C]MET (4.27 ± 1.91) was significantly lower compared to [18F]FDG (13.94 ± 5.65) (*p* < 0.002). No differences in terms of the tumor-to-normal-tissue ratio (T/N) were reported between the two tracers, while significant correlations were demonstrated for SUV and T/N for the two modalities (r 0.62 and r 0.63, respectively, *p* < 0.03).

### 3.3. Therapy Response Assessment and Prognostic Role of [11C]MET PET Imaging in Lymphomas

At first, the role of [11C]MET PET in the response assessment of PCNSL patients treated with radiation therapy was assessed by Ogawa et al. [45]. The authors reported higher tracer uptake of all lesions before therapy: The extension corresponded to CT or MRI findings for 5/7 subjects, while in the remaining two patients, the extension of PET uptake was higher compared to conventional imaging findings. [11C]MET PET also revealed tracer uptake in a patient with secondary CNS lymphoma. The size and degree of uptake were reduced after or during radiotherapy in seven PET scans, while in eight scans, the extent of increased uptake was larger compared to CT or MRI findings and, interestingly, one of these patients had tumor recurrence in the site of residual tracer uptake. Additionally, the differential absorption ratio (DAR) between the neoplastic lesion and the controlateral posterior temporal gray matter decreased significantly after the completion of therapy (*p* < 0.05).

As previously mentioned, Kaste et al. [53] compared [18F]FDG and [11C]MET PET/CT in the assessment of pediatric lymphomas. At the restaging evaluation, they reported concordant uptakes between the two tracers for 14 patients, with positive scans confirmed in four subjects and the resolution of uptakes in 10 patients. In one patient, metabolic activity was minimally discordant between the two modalities, with normalization of [18F]FDG distribution and slightly positivity at [11C]MET; however, at the last follow-up, the patient was alive and without relapse of disease.

Miyakita et al. [60] reported that the optimal cut-off value for [11C]MET T/N to differentiate between complete response and persistence or progression of PCNSL after therapy was 1.83, with an area under the curve (AUC) of 0.951, a sensitivity of 82.4% and a specificity of 100%. Additionally, a cut-off of 1.80 (AUC 0.932, sensitivity 85.3% and specificity 85%) was the best discriminator between persistence/progression of disease and the presence of small enhancing lesions at MRI, defined as unconfirmed complete response.

An interesting paper by Ahn et al. [54] investigated the prognostic role of [11C]MET PET imaging in PCNSL, revealing no differences in initial metabolic tumor volume (MTV) and T/N between the different risk classes; however, higher risk groups had higher values at the interim evaluation. Additionally, at the interim evaluation performed after chemotherapy, MRI and PET/CT were discordant in 4/26 of the subjects, with negative [11C]MET imaging and the presence of remnant signals at MRI; however, all of these patients achieved complete remission after primary treatment. Interim semiquantitative parameters revealed the ability to predict disease progression with an AUC of 0.804 (sensitivity, 71.4%, specificity 83.3%; *p* < 0.001) for a value of 1.67 for T/N and an AUC of 0.786 (sensitivity 85.7%, specificity 66.7%; *p* 0.014) for a value of 0.321 for MTV, while these parameters failed to predict overall survival (OS). For progression free survival (PFS), the positive predictive values for the interim T/N ratio and MTV were 80.7% and 72.3%, respectively. In the univariate analysis, high interim T/N ratio, high MTV and high CSF protein level had significant predictive value for PFS (*p* < 0.05), while only a high T/N and high CSF protein level were significant predictors of OS (*p* < 0.05). In the multivariate analysis, a high interim T/N was an independent prognosticator for PFS (*p* 0.044) and OS (*p* 0.043). Lastly, the study previously described and performed by Nuutinen et al. [47] revealed that patients’ survival did not correlate with [11C]MET uptake considering both SUV or K_i_.

### 3.4. [11C]MET PET in the Differential Diagnosis of Lymphomas and Other Pathological Conditions

Okada et al. [52] evaluated the role of [11C]MET and [18F]FDG PET in the differential diagnosis of newly diagnosed intracranial DLBCL and glioblastoma multiforme (GBM). The authors reported that the homogeneous uptake of both tracers was present for 6/7 DLBCL patients, while heterogeneous patterns were reported for both tracers in all 15 GBM. No significant differences in terms of SUVmax at early or late phases were reported at [11C]MET PET between the two conditions. In addition, the ratio of SUVmax between late and early phase (ΔSUVmax) at [11C]MET was significantly higher for GBM, and when the highest value (1.17) was used as a cutoff point to distinguish between the two pathological entities, the sensitivity and the specificity were 100%. As a confirmation, AUC were 0.621, 0.714 and 1.000 for SUVmax in the early phase, SUVmax in the late phase and ΔSUVmax, respectively.

A similar research was performed by Postnov et al. [55] to investigate the ability of [18F]FLUDA and [11C]MET PET/CT to make a differential diagnosis between PCNSL and GBM. All the tumors, even when considering PCNSL under corticosteroid therapy, displayed a pronounced uptake of [11C]MET; however, this was without significant differences in terms of T/N and SUV. The ΔSUVmax was significantly different between PCNSL and GBM (1.06 ± 0.07 g/mL for GBM and 1.18 ± 0.05 g/mL for PCNSL, *p* 0.001), resulting in an AUC of 0.9 with a cutoff of 1.10 (100% sensitivity and 80% specificity). In addition, averaged T/N and ΔSUVmax curves demonstrated a clear difference in [11C]MET uptake between GBM and PCNSL, at both early and late time points. Despite these findings, the authors underlined that [18F]FLUDA had a clear difference in dynamic uptake between GBM and PCNSL, since the first one had a decrease over time after an early maximum, while the latter had a steady increase over time. This tracer was therefore shown to likely be a promising radiopharmaceutical for differentiating PCNSL from other malignancies.

Similarly, Inoue et al. [56] compared different preoperative quantitative indicators for the differential diagnosis of GBM and PCNSL, revealing that [11C]MET SUVmax and T/N were high in both diseases, again without significant differences among them. Interestingly, [18F]FDG T/N was significantly different between the two conditions and had high sensitivity and specificity in the differential diagnosis between them.

A comparison between [11C]MET and [18F]FDG PET/CT in distinguishing between PCSNL and isocitrate dehydrogenase wildtype glioblastoma was performed by Norikane et al. [58]. The authors reported that at qualitative analysis, all neoplasms (22 PCNSL and 64 glioblastoma) had [11C]MET uptake, while [18F]FDG was positive in 95% of PCNSL and 84% of glioblastomas. No differences in terms of T/N at [11C]MET were reported (*p* 0.37), while T/N at [18F]FDG was significantly higher for PCNSL (*p* < 0.001). In addition, AUC of [18F]FDG T/N was significantly higher than AUC of [11C]MET T/N (0.871 versus 0.565, respectively, *p* 0.003).

Leskinen Kallio et al. [44] performed a study to analyze the added value of [11C]MET PET in the assessment of head and neck cancers, also including eight patients with lymphoma. All lymphomas were clearly visible at PET evaluation and the uptake of [11C]MET was somewhat higher in the squamous cell carcinoma compared to lymphomas. Similarly, Aki et al. [51] analyzed different brain tumors with dynamic [11C]PET, also including 14 lymphomas, and revealed that their uptake increased significantly with time (*p* < 0.05), similarly to GBM and differently from meningiomas or oligodendrogliomas.

The ability of [11C]MET PET to make differential diagnosis between different brain neoplastic conditions was also assessed by Nomura et al. [59]. They reported that PCNSL had a typical time–activity curve, characterized by an SUV with a low level in the initial phase, a rapid increase in the early phase and a continuous increase in the late phase. The time-to-peak (TTP) was significantly longer in PCSNL compared to GBM (*p* < 0.01) while the slope of the curve in the late phase was significantly higher for PCNSL compared to GBM (*p* < 0.01). A cut-off value of 27.5 min for TTP had a 100% sensitivity and a 54.8% specificity in the differential diagnosis between these two conditions with an AUC of 0.819, while a cut-off of 0.54h-1 for the slope value had a sensitivity of 100% and a specificity of 67.3% with an AUC of 0.862.

Lastly, Ohmura et al. [57] investigated the role of [11C]MET PET/CT in differentiating brain lesions with a similar appearance at conventional CT and MRI, also including 15 PCNSL. The authors based their analysis on five different PET parameters such as higher T/N (H), overextension beyond gadolinium (O), peripheral pattern (P), central pattern (C) and dynamic-up (D). For the H parameter, significant differences were reported between PCNSL and tumefactive multiple sclerosis (*p* 0.01), even though, after multiple-comparison correction, the significance was not confirmed. For the O parameter, there was a significantly different incidence between GBM and PCNSL (*p* < 0.001) and between PCNSL and metastatic brain tumor (*p* 0.04). The incidence of the P parameter was significantly different between GBM and PCNSL (*p* < 0.001), while the C parameter was significant different between GBM and PCNSL (*p* < 0.001), between PCNSL and tumefactive multiple sclerosis (*p* 0.01) and between PCNSL and radiation necrosis (*p* 0.01), and tended to be only markedly higher for PCNSL compared to metastatic brain tumor (*p* 0.05). The incidence of the D parameter was significantly different between GBM and PCNSL (*p* 0.01) and tended to be markedly higher than metastatic brain tumor (*p* 0.05). In addition, the use of different combinations of sets of two of these diagnostic features revealed that all the combinations had an AUC that ranged from 0.85 to 1.00.

## 4. Discussion

In general, [11C]MET PET imaging has demonstrated its ability to assess the presence of lymphomas since they exhibit high uptake of this particular tracer. This is true when considering both lymphomas from all the body or when focusing only on lymphomas of the central nervous system. In the first case, intense uptake of the tracer was demonstrated in most of the patients and in most of the anatomical regions affected by the disease [44,46,47,48,53]. Interestingly, different studies compared [11C]MET and [18F]FDG results, revealing some discordant results: In some cases, the uptake of the first tracer was higher than that of the second; in other papers, the ability of the two tracers was defined as comparable and, lastly, a study revealed the ability of [18F]FDG to reveal one third more foci of disease. In this scenario, it was also suggested that physiological accumulation of [11C]MET hampered more the interpretation of the images compared to [18F]FDG, while [11C]MET could be preferable in the staging setting in hyperglycemic patients [44,46,48,53].

Another interesting point that several papers have investigated is the ability of PET findings to differentiate between low- and high-grade lymphomas. Again, heterogeneous findings were reported in this setting since, in most of the cases, no significant differences in terms of uptake or semiquantitative parameters were demonstrated between different grades, while a single study revealed that Ki was significantly higher in high grade lymphomas [44,46,47,48,53]. Additionally, it was also reported that lymphomas with higher Ki and uptake had a higher amount of cells in the S-phase, a finding not confirmed with [18F]FDG [44,47]. One of the issues that is worth underlining and that can affect the review is the fact that two of the papers included focused on “intermediate grade lymphomas”, as specified by the authors [43,47]. This could be a problem since these papers have been published in the 1900s and, nowadays, the grading classification of lymphomas has changed, dividing these pathologies into low- and high-grade neoplasms. This modern subdivision has a clinical impact, given the fact that high-grade malignancies are characterized by worst outcomes in prognostic terms when compared to low-grade forms. Additionally, the possibility of a low-grade lymphoma to transform into a high-grade pathology is well known and [18F]FDG PET imaging has demonstrated its ability to detect this transformation based on the degree of tracer uptake since indolent forms may express low uptake [61,62].

As previously mentioned, [11C]MET PET is a useful tool for the assessment of the central nervous system given its low physiological uptake in this region and, therefore, different papers focused their analyses on PCNSL. These studies were performed by comparing the diagnostic ability of [11C]MET and [18F]FDG, revealing similar and high sensitivity for both the tracers. In addition, SUVmax for the two modalities and [18F]FDG kinetics parameters were demonstrated as significantly higher in diseases with typical appearance at MRI compared to those with atypical appearance [49,50].

The value of [11C]MET PET imaging for the evaluation of lymphomas in a restaging setting has been also studied in several papers. Most of them focused their research on PCNSL and revealed that this imaging modality had the ability to assess the response to therapy, with a reduction in the size and/or degree of tracer uptake after its completion. Interestingly, T/N had a high AUC in the differential diagnosis between complete response and persistence or progression of disease and, moreover, a single paper revealed that cases with discordant findings between negative PET and remnant at MRI achieved complete remission [45,54,60]. In addition, a single paper focusing on pediatric lymphomas reported that in most of the patients, both [11C]MET and [18F]FDG PET PET/CT were concordant in the post-therapeutic evaluation [53]. Furthermore, in prognostic terms, a study revealed that interim T/N at [11C]MET imaging was an independent prognosticator for both OS and PFS, while, in contrast with these findings, another paper revealed that patients’ survival did not correlate with [11C]MET uptake [47,54].

The ability of [11C]MET PET imaging to support a differential diagnosis between lymphomas and other different pathological conditions has been investigated in several papers and all of them focused their attention on the head and neck region. In fact, some papers revealed no significant differences in terms of semiquantitative parameters between PCNSL and GBM or isocitrate dehydrogenase wildtype glioblastoma, while [18F]FDG T/N demonstrated the ability to differentiate between these two conditions with high sensitivity and specificity [51,56,58]. In contrast with these findings, it was also reported that PCNSL had a typical dynamic pattern of uptake at [11C]MET PET that was significantly different from the GBM pattern and, moreover, some different parameters of uptake were significantly differently between the two conditions, but also between PCNSL and metastatic brain tumor as well as tumefactive multiple sclerosis and radiation-induced necrosis [57,59]. Similarly, it was reported that ΔSUVmax was significantly higher in GBM compared to intracranial DLBCL [52]. Moreover, a single comparison between [11C]MET and [18F]FLUDA PET imaging revealed that the first modality had the ability to differentiate between PCNSL and GBM with a 100% sensitivity and an 80% specificity [55]. Lastly, some insights on the ability of [11C]MET PET imaging to differentiate between lymphomas and different head and neck cancers have been reported [44,51].

As mentioned, [18F]FDG PET is the nuclear medicine imaging modality that has proven its role in the assessment of lymphoma, becoming, therefore, a pivotal modality for the evaluation of both HL and NHL. In this scenario, a clear added value of [11C]MET over [18F]FDG PET imaging has not been underlined in this review, since in most of the settings evaluated the two tracers revealed similar results. However, a possible application of the first modality could be the staging setting of hyperglycemic patients, having a well-known condition able to hamper the use of [18F]FDG and its diagnostic accuracy [63,64]. Furthermore, some studies included in the review reported the high sensitivity of [11C]MET PET for the assessment of PCNSL, a fact that could endorse the use of this tracer in this setting, since the physiological uptake of [18F]FDG by the brain could reduce its diagnostic accuracy [65]. As previously underlined, it is, however, worth underlining that these two tracers reflect distinct metabolic pathways, and maybe, an integration of the information obtained for both of them could be the best approach in some specific and selected cases. Further research in this scenario is, however, mandatory to correctly define the added value of [11C]MET over [18F]FDG. PET is a well-established imaging modality that plays an important role in the assessment of a high number of different diseases. Generally speaking, the different radiopharmaceuticals commonly used have not demonstrated particular side effects; therefore, in this setting [11C]MET seems not to have a particular advantage over [18F]FDG. It is, however, worth underlining that the half-life of [11C] is approximately 20 min, which, if compared to the approximately 110 min of [18F], is a characteristic that should be taken into account in the daily clinical practice. To sum up, when comparing [11C]MET and [18F]FDG, the strengths of the first tracer are the fact that it is able to image different metabolic pathways compared to the second, its ability to investigate regions where a physiological uptake of [18F]FDG could hamper their evaluations (such as in PCSNL) and the fact that its uptake is nondependent on the glycemia of the patient. Conversely, the limitations of the use of [11C]MET imaging could be related to its shorter half-life if compared to [18F]FDG, its availability for the daily clinical practice and the fact that the second tracer has a clear and established prognostic value.

Some limitations derived from the characteristics of the papers included in the review clearly affect our findings. First, most of the studies are characterized by small and heterogeneous cohorts. In addition, different types of lymphomas affecting different regions are included in the present review. Consequently, the descriptive analyses that were performed are based on limited samples. Moreover, most of the papers had a retrospective design. Based on these facts, no meta-analysis of the data retrieved could be performed. As a consequence, even though our findings revealed a possible role for [11C]MET PET imaging for the assessment of lymphomas in different clinical settings, new efforts and larger prospective researches are required to clearly assess and confirm this fact.

## 5. Conclusions

In conclusion, even with heterogeneous evidence, this review revealed a possible role for [11C]MET PET imaging in the assessment of lymphomas affecting both the whole body and the central nervous system. These insights were reported in different settings, such as the staging of the disease, the evaluation of the response to therapy and differential diagnosis with different pathological conditions affecting the head and neck region (Table 4). When compared to [18F]FDG imaging, in general, similar results have been reported between the two modalities in these settings.

## Figures and Tables

**Table 1:** Exclusion and inclusion criteria used to include papers in the systematic review.

Exclusion	Inclusion
Preclinical studies Conference proceedings Reviews Editorials Only 1 lymphoma patient imaged with [11C]MET PET	English language

[11C]MET PET: [11C] methionine positron emission tomography.

**Table 2:** Characteristics of the studies considered for the review.

First Author	N. Ref.	Year	Country	Study Design	N. Pts (with Lymphoma)	CNSL
Leskinen-Kallio S	[43]	1991	Finland	Prospective	14 (14)	No
Leskinen-Kallio S	[44]	1994	Finland	Prospective	47 (8)	No
Ogawa T	[45]	1994	Japan	ns	10 (10)	Yes
Rodriguez M	[46]	1995	Sweden	Prospective	23 (23)	No
Nuutinen J	[47]	1998	Finland	Retrospective	32 (32)	No
Sutinen E	[48]	2000	Finland	Prospective	19 (19)	No
Kawai N	[49]	2010	Japan	Retrospective	17 (17)	Yes
Kawase Y	[50]	2010	Japan	Retrospective	13 (13)	Yes
Aki T	[51]	2012	Japan	Retrospective	144 (14)	Yes
Okada Y	[52]	2012	Japan	Retrospective	22 (7)	Yes
Kaste SC	[53]	2017	USA	Prospective	18 (18)	No
Nomura Y	[59]	2018	Japan	Retrospective	160 (8)	Yes
Ahn SY	[54]	2018	Korea	Prospective	26 (26)	Yes
Miyakita Y	[60]	2020	Japan	Retrospective	36 (36)	Yes
Postnov A	[55]	2022	France	Prospective	31 (18)	Yes
Inoue A	[56]	2023	Japan	Retrospective	96 (68)	Yes
Ohmura K	[57]	2023	Japan	Retrospective	129 (15)	Yes
Norikane T	[58]	2024	Japan	Retrospective	86 (22)	Yes

N.: number; Pts: patients; Ref.: reference; ns: not specified; CNSL: central nervous system lymphoma; USA: United States of America.

**Table 3:** Results and main findings of the studies considered for the review.

First Author	Device	Tracer	Reported Mean Activity (MBq)	PET Analysis	Setting	Main Findings
Leskinen-Kallio S [43]	PET	[11C]MET, [18F]FDG	125–300 for [11C]MET, 230–340 for [18F]FDG	Qualitative and semiquantitative	Assessment of disease	[11C]MET uptake rate was significantly higher than [18F]FDG. [18F]FDG accumulated clearly in high and intermediate lymphomas, while all but 1 had [11C]MET uptake. Tumor/plasma ratio increased faster in higher grade NHL for both tracers even with considerable overlap between grades.
Leskinen-Kallio S [44]	PET	[11C]MET	273.8 ± 59.2	Qualitative and semiquantitative	Differential diagnosis	Lymphomas were clearly visible at PET imaging, even though uptake was lower than squamous cell carcinoma.
Ogawa T [45]	PET	[11C]MET	740–1480	Qualitative and semiquantitative	Response assessment and prognosis	PET clearly depicted CNS lymphomas before RTT and the extent of uptake decreased after therapy. A patient was confirmed to have recurrence after PET images.
Rodriguez M [46]	PET	[11C]MET, [18F]FDG	800 for [11C]MET, 400 for [18F]FDG	Qualitative and semiquantitative	Assessment of disease	All tumors had uptake of both tracers. [18F]FDG had the ability to discriminate between high and low grade lymphomas, while [11C]MET did not.
Nuutinen J [47]	PET	[11C]MET	293 (125–537)	Qualitative and semiquantitative	Assessment of disease and prognosis	[11C]MET PET had a high sensitivity for the detection of lymphoma and could differentiate between high and low grade. [11C]MET uptake did not predict survival.
Suutinen E [48]	PET	[11C]MET, [18F]FDG	439 (321–478) for [11C]MET, 370 (292–395) for [18F]FDG	Qualitative	Assessment of disease	[18F]FDG and [11C]MET seemed to be comparable in the detection of lymphoma. Physiological accumulation of [11C]MET seemed to hamper the evaluation of images; however, this tracer may be preferable in hyperglycemic patients.
Kawai N [49]	PET	[11C]MET, [18F]FDG	142–321 for [11C]MET, 114–267 for [18F]FDG	Qualitative and semiquantitative	Assessment of disease	Typical primary CNS lymphoma showed strong uptake of both tracers, while visual analysis for atypical forms was not useful. SUVmax and influx rate of [18F]FDG were significantly lower in atypical forms. K3 values were similar for typical and atypical CNS lymphomas.
Kawase Y [50]	PET	[11C]MET, [18F]FDG	6/kg for [11C]MET, 4.5/kg for [18F]FDG.	Qualitative and semiquantitative	Assessment of disease	No significant differences of T/N between [11C]MET and [18F]FDG, although uptake of the first tracer was significantly lower than [18F]FDG.
Aki T [51]	PET	[11C]MET	6.2–7.4/kg	Qualitative and semiquantitative	Differential diagnosis	Significant dynamic increase of the maximum [11C]MET T/N was seen in glioblastomas and malignant lymphomas.
Okada Y [52]	PET	[11C]MET, [18F]FDG	11.1/kg for [11C]MET, 3.7/kg for [18F]FDG	Qualitative and semiquantitative	Differential diagnosis	[18F]FDG SUVmax was significantly higher for DLBCLs compared to GBM. SUVmax in the late and early phases of [11C]MET PET was not significantly different between the two conditions; however, the values of ΔSUVmax on MET PET in DLBCL were significantly higher than those in GBM.
Kaste SC [53]	PET/CT	[11C]MET, [18F]FDG	740/1.7 m^2^ for [11C]MET, 5.5/kg for [18F]FDG	Qualitative and semiquantitative	Assessment of disease and response assessment	[11C]MET is elevated in most regions involved at diagnosis and follow-up. At baseline, all nodal groups demonstrated concordant [11C]MET and [18F]FDG uptake, except for 3 groups that were Waldeyer’s ring, paraaortic region and liver. Normal intense [11C]MET uptake in the pancreas and liver reduced sensitivity for disease detection in these regions.
Nomura Y [59]	PET	[11C]MET	3.5/kg	Qualitative and semiquantitative	Response assessment and prognosis	Quantification of the time–activity curve in different brain tumors identified by a dynamic [11C]MET PET could be helpful in the non-invasive differential diagnosis.
Ahn SY [54]	PET/CT	[11C]MET	7/kg	Qualitative and semiquantitative	Response assessment and prognosis	Higher International Extranodal Lymphoma Study Group risk scores were associated with higher interim MTV and T/N. Interim MTV and T/N ratios predicted PFS and OS, respectively. High interim T/N was associated with decreased PFS.
Miyakita Y [60]	PET/CT	[11C]MET	4/kg	Qualitative and semiquantitative	Differential diagnosis	A T/N ≥ 1.80 could help in the detection of active PCNSL after treatment; therefore, [11C]MET PET may be a useful tool for accurate evaluation of the treatment efficacy in these neoplastic conditions.
Postnov A [55]	PET/CT	[11C]MET, [18F]FLUDA	3.22 ± 0.5/kg for [11C]MET, 4.05 ± 0.26/kg for [18F]FLUDA	Qualitative and semiquantitative	Differential diagnosis	No significant differences in [11C]MET uptakes were observed among PCNSL and GBM. Difference in dynamic [18F]FLUDA uptake was observed for GBM.
Inoue A [56]	ns	[11C]MET, [18F]FDG	5/kg for [11C]MET, 3.5/kg for [18F]FDG	Qualitative and semiquantitative	Differential diagnosis	T/N ≥ 2.4 on [18F]FDG PET was quite specific for PCNSL. No other examinations displayed any significant differences between PCNSL and GBM.
Ohmura K [57]	PET/CT	[11C]MET	3.5/kg	Qualitative and semiquantitative	Differential diagnosis	Five diagnostic criteria obtained from [11C]MET PET imaging could make differential diagnosis between five types of brain lesions. Differences in 5 diagnostic variables were unique to each of the 5 lesions.
Norikane T [58]	PET/CT	[11C]MET, [18F]FDG	6/kg for [11C]MET, 3.7/kg for [18F]FDG	Qualitative and semiquantitative	Differential diagnosis	All PCNSLs and glioblastomas were [11C]MET positive, while 95% and 84% were respectively [18F]FDG positive. No difference in [11C]MET T/N between PCNSL and glioblastoma was reported, while [18F]FDG T/N was significantly higher in PCNSL. AUC value was significantly higher for the [18F]FDG T/N ratio.

[11C]MET: [11C]methionine; [18F]FDG: 18F-fluorodesoxyglucose; [18F]FLUDA: 18F-fludarabine; ns: not specified; PET: positron emission computed tomography; CT: computed tomography; MBq: megabecquerel; kg: kilogram, SUVmax: standardized uptake value; CNS: central nervous system; RTT: radiotherapy; DLBCL: diffuse large B-cell lymphoma; T/N: tumor-to-normal-tissue ratio; OS: overall survival; PFS: progression free survival; MTV: metabolic tumor volume; BGM: glioblastoma multiforme; AUC: area under the curve; ΔSUVmax: ratio of SUVmax between late and early phase.

**Table 4:** Key findings and evidence gaps on the role of [11C]MET PET imaging for the assessment of lymphomas.

Key Findings	Evidence Gaps
Lymphomas exhibit [11C]MET uptake [11C]MET is particularly useful to assess regions with high physiological uptake of [18F]FDG and in hyperglycemic subjects [11C]MET can assess response to therapy, in particular for PCNSL [11C]MET could have a role in the differential diagnosis between lymphomas and other pathological conditions	Discordant results comparing [11C]MET and [18F]FDG diagnostic abilities Heterogeneous findings for [11C]MET in the differential diagnosis between high- and low-grade lymphomas Limited and contrasting insights on the prognostic value of [11C]MET

[11C]MET: 11C-methionine; [18F]FDG: 18F-fluorodesoxyglucose; PCNSL: primary central nervous system lymphomas.

## Data Availability

Data supporting the reported results can be found using the public PubMed/MEDLINE, Scopus and Cochrane Library databases.
